# Ring opening polymerization of d,l-lactide and ε-caprolactone catalysed by (pyrazol-1-yl)copper(ii) carboxylate complexes†

**DOI:** 10.1039/d1ra00339a

**Published:** 2021-04-12

**Authors:** Divambal Appavoo, Lara C. Spencer, Ilia A. Guzei, Carlos J. Gómez-García, Juanita L. van Wyk, James Darkwa

**Affiliations:** Department of Chemical Sciences, University of Johannesburg P.O. Box X524, Auckland Park, 2006 South Africa jdarkwa@gmail.com; Department of Chemistry, University of Wisconsin-Madison Madison WI 53706 USA; Department of Inorganic Chemistry and ICMol, C/ Catedrático José Beltrán, 2. University of Valencia 46980 Paterna Valencia Spain

## Abstract

1,2-Bis{(3,5-dimethylpyrazol-1-yl)methyl}benzene (L) reacts with [Cu(OAc)_2_] and C_6_H_5_COOH, 4-OH-C_6_H_4_COOH, 2-Cl-C_6_H_4_COOH and (3,5-NO_2_)_2_-C_6_H_3_COOH to afford the copper complexes [Cu_2_(C_6_H_5_COO)_4_(L)_2_] (1), [Cu_2_(4-OH-C_6_H_4_COO)_4_(L)_2_] (2), [Cu_2_(2-Cl-C_6_H_4_COO)_4_(L)_2_]_*n*_ (3) and [Cu{(3,5-NO_2_)_2_-C_6_H_3_COO}_2_L]_*n*_ (4) which are characterised by IR, mass spectrometry, elemental analyses, and X-ray crystallography. The structural data revealed two geometries that are adopted by the complexes: (i) paddle wheel in 1, 2·7H_2_O, 3 and (ii) regular chains in 3 and 4. Magnetic studies show strong antiferromagnetic couplings in the paddle wheel complexes and a weak antiferromagnetic coupling in the monometallic chain one. Catalysis studies performed with these complexes (1–4) showed that they initiate ring opening polymerization (ROP) of ε-caprolactone (ε-CL) under solvent-free conditions and d,l-lactide in toluene at elevated temperatures. Polycaprolactone (PCL) and poly(d,l-lactide) (PLA) obtained from the polymerization reactions are of low molecular weights (858 for PCL and 602 Da for PLA for initiator 1) and polydispersity indices (typically 2.16 for PCL and 1.64 for PLA with 1 as the initiator). End group analysis of the polymers, determined by MALDI-ToF MS, indicates that the polymers have benzoate, hydroxyl, methoxy and cyclic end groups.

## Abstract 2

We report the synthesis, structure and complete characterization of four new pyrazolyl carboxylate-based copper(ii) complexes that catalyze the ring opening polymerization of ε-caprolactone under solvent-free conditions and of d,l-lactide in toluene.

## Graphical abstract

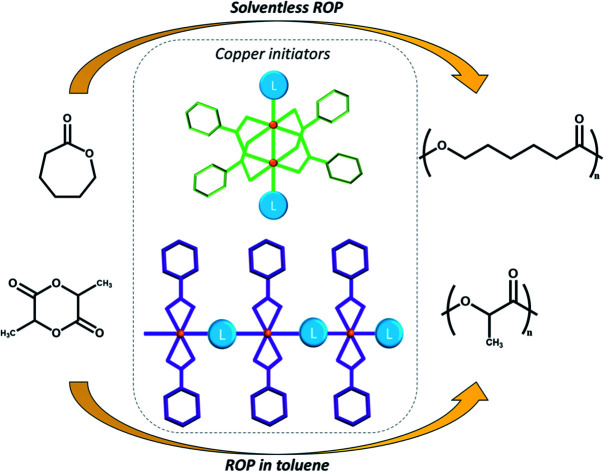

## Introduction

Aliphatic polyesters continue to receive much interest due to their unique biodegradability, biocompatibility and permeable properties that make them very useful in the medical, pharmaceutical and environmental fields.^1–5^ The most convenient method for preparing these polyesters is *via* ring-opening polymerization (ROP) of the respective cyclic esters. The key to achieving controlled ROP, in terms of molecular weight distribution and stereochemistry, lies in the performance of the initiator or catalyst. Different types of catalysts have been studied for this purpose, including organometallic compounds, organocatalysts^6,7^ as well as enzymes.^8^ Some common initiators or catalysts reported are [Al(O^*i*^Pr)_3_]^9,10^ and [Sn(Oct)_2_].^1,11^ However, due to drawbacks associated with these aluminium and tin catalysts, such as low conversions, poor selectivity and backbiting reactions, the search for better catalysts continues.

Several metal-based catalysts have been studied, ranging from main group elements as Ca,^12^ Al,^13^ Li,^14–16^ Mg,^17–20^ Sn^21^ to transition metals such as Fe,^22–24^ Zn,^25^ Zr,^26–28^ and lanthanoids.^29^ One metal of growing interest is copper, which although has not been investigated as much as metals like zinc, shows interesting properties such as biocompatibility and good electron transfer ability that make Cu-based complexes good candidates for ROP catalysis.^30–32^

The catalytic activity of organometallic complexes is largely determined by the ligands attached to the metal centre. For instance, backbiting reactions can be eliminated by using a suitable sterically bulky ligand that provides some amount of steric barrier around the metal centre. Several ligands have been explored to obtain the optimum catalytic performance of metal complexes; including amino-phenolate,^33–35^ salicylaldimine,^32^ formamide,^36,37^ pyrazolyl,^30,38–41^ and benzoimidazolyl.^42–44^ Pyrazole and its derivatives constitute an important group of N-donor ligands that have been used because of their attractive coordination chemistry.^45^ The structural flexibility of pyrazole and pyrazolyl ligands constitute an interesting advantage. Furthermore, the denticity of pyrazolyl ligands can be greater or equal to one, and the steric hindrance of a pyrazolyl ligand can be controlled by the appropriate choice of groups on the pyrazole ring.^40,46–48^

We previously reported the ROP of ε-caprolactone (ε-CL) and d,l-lactide using bis(3,5-dimethylpyrazole)–Zn(ii) and Cu(ii) complexes as initiators.^41^ The current report further explores the initiator systems that consist of the bidentate ligand, 1,2-bis((3,5-dimethylpyrazol-1-yl)methyl)-benzene (L) coordinated to copper through the pyrazolyl nitrogen atoms, and ancillary ligands derived from a range of benzoic acids, also coordinated to the copper through carboxylate oxygen atoms. Such copper complexes usually adopt a paddle-wheel arrangement, with four carboxylates in equatorial positions that bridge two copper centres and the N-donor ligand occupying the axial positions. Depending on the carboxylate, the coordination geometry around the copper centre can vary, hence impacting the magnetic and electronic properties of the complex. We describe herein the coordination chemistry of four pyrazolyl copper carboxylate complexes formulated as [Cu_2_(C_6_H_5_COO)_4_(L)_2_] (1), [Cu_2_(4-OH-C_6_H_4_COO)_4_(L)_2_] (2), [Cu_2_(2-Cl-C_6_H_4_COO)_4_(L)_2_]_*n*_ (3) and [Cu{(3,5-NO_2_)_2_-C_6_H_4_COO}_2_L]_*n*_ (4). Three of them show the classical paddle-wheel structure (1–3) and the fourth one is monometallic but polymeric with monodentate L linkages (4). We report their magnetic properties and their structure–activity relationship in initiating the ROP of d,l-lactide and ε-caprolactone.

## Results and discussion

### Synthesis and spectral characterization of complexes

**Scheme 1:**
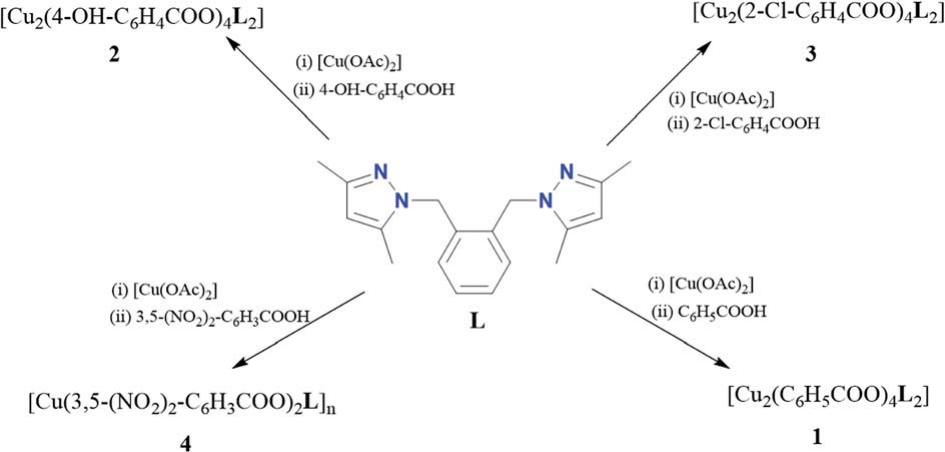
Synthesis of complexes 1–4.

Complexes 1–4 were synthesized from one-pot reactions of copper(ii) acetate [Cu(OAc)_2_] with different benzoic acids, namely (C_6_H_5_COOH (benzoic acid), 4-OH-C_6_H_4_COOH (4-hydroxybenzoic acid), (3,5-NO_2_)_2_-C_6_H_3_COOH (3,5-dinitrobenzoic acid) and 2-Cl-C_6_H_4_COOH (2-chlorobenzoic acid) and 1,2-bis((3,5-dimethylpyrazol-1-yl)methyl)benzene (L) (Scheme 1). The reactions proceeded *via* an initial substitution of the acetate in the copper starting complex by the benzoate group of the respective benzoic acid, accompanied by the release of acetic acid. The appropriate copper benzoate then reacted with L and produced the corresponding pyrazolyl copper benzoate complexes (1–4) in moderate yields (57–78%). Complexes 1–3 were obtained as green solids, while complex 4 was isolated as a purple solid.

Complexes 1–4 were characterized by IR spectroscopy, mass spectroscopy and elemental analysis. Formation of the complexes was supported by IR data that showed shifts in the *ν*_C=O_ band when compared to the corresponding unbound benzoic acid. For example, for complex 3, the IR spectrum shows the carbonyl peak at 1631 cm^−1^, a significant shift from 1591 cm^−1^ in the free benzoic acid. This shift is accompanied by the disappearance of the *ν*_O–H_ band of the benzoic acid in the region of 2900 cm^−1^, further supporting complexation of the benzoate to the metal.

Mass spectra data provide additional evidence of the formation of the complexes. For instance, 2 showed a peak at *m*/*z* = 494 corresponding to the fragment of the molecular ion after loss of one benzoate group. Similar mass spectra were obtained for the other complexes, with peaks associated with the parent compound without one benzoate. The purities of the complexes were determined by elemental analysis and good correlations between the calculated and found values were obtained.

### X-ray structures of complexes

**Fig. 1:**
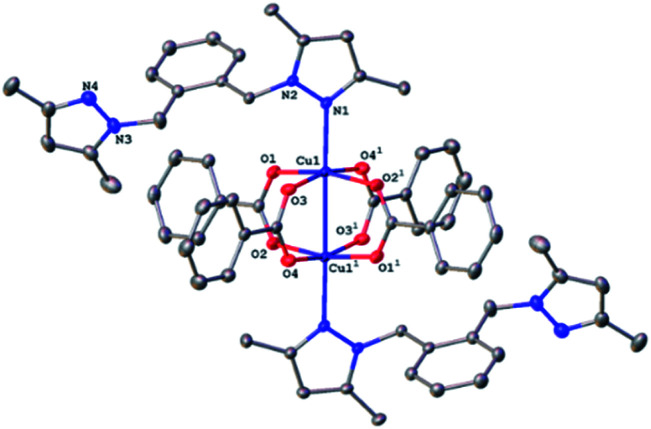
A molecular drawing of complex 1 shown with 50% probability ellipsoids. All H atoms are omitted. Symmetry code: (1) −*x*, −*y*, −*z*.

**Fig. 2:**
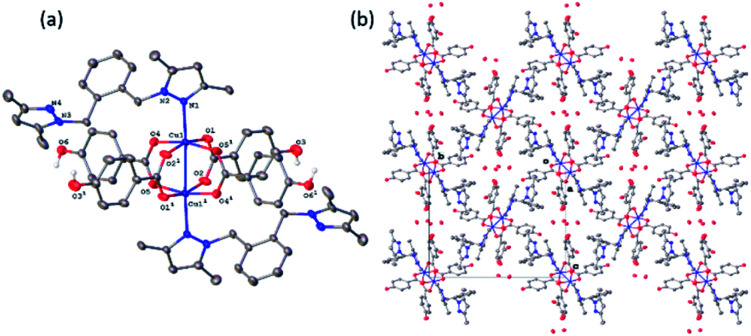
(a) A molecular drawing of complex 2 shown with 50% probability ellipsoids. All solvent water molecules and all H atoms attached to C atoms are omitted. Symmetry code: (1) 1 − *x*, −*y*, 2 − *z*. (b) A packing diagram of complex 2 viewed along the *a* axis illustrates the solvent-accessible voids in the lattice. The red isolated atoms represent the solvent water molecules in these voids. All H atoms are omitted.

**Fig. 3:**
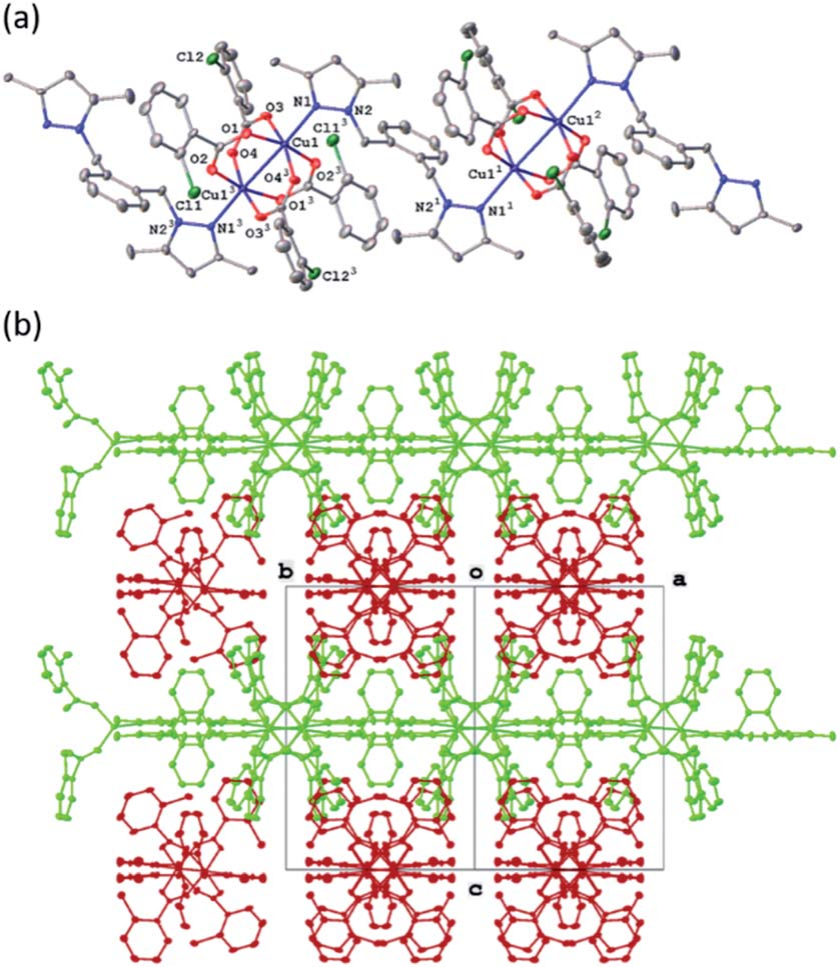
(a) A molecular drawing of complex 3 shown with 50% probability ellipsoids. Symmetry codes: (1) 0.5 − *x*, −0.5 − *y*, *z*; (2) −1/2 + *x*, −1/2 + *y*, −*z*; (3) 1 − *x*, −*y*, −*z*. (b) A packing diagram of complex 3 viewed along the [110] direction. The molecules are coloured red and green to illustrate the two directions of propagation of the polymeric chains. Red chains propagate in the [110] direction whereas the green ones in the [110] direction. All H atoms are omitted in (a) and (b).

**Fig. 4:**
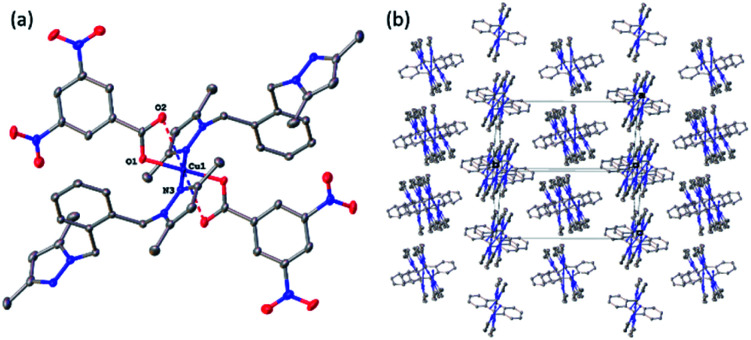
(a) A molecular drawing of complex 4 shown with 50% probability ellipsoids. Full coordination sphere of atom Cu1 is shown with selected symmetry-independent atoms labelled. (b) A packing diagram of complex 4 showing the polymeric chains. The projection is slightly off the [101] direction to show the chains. The carboxylates have been omitted for clarity.

All four copper complexes were characterized by single-crystal X-ray diffraction; their molecular geometries are presented in Fig. 1–4 and Table S1.† The four complexes show structural diversity in the solid state: compounds 1–3 are dimeric paddle-wheel complexes^49–52^ with weak cuprophilic interactions between octahedral copper centres. Compound 1 crystallizes with discreet dimeric complexes (Fig. 1). Complex 2 co-crystallizes with ∼7 water molecules per dimer (Fig. 2a) and all entities form hydrogen bonds resulting in a three-dimensional hydrogen-bonded framework (Fig. 2b). Complex 3 is a one-dimensional pyrazole-carboxylate coordination polymer connecting dimeric Cu complexes (Fig. 3). Compound 4 is also polymeric (Fig. 4), however the metal core is mononuclear and the Cu centre is square bipyramidal. All bidentate pyrazole-containing linkers have one pyrazole coordinated to a Cu centre whereas the unsubstituted N atom of the pyrazole unit on the other end of the ligand can either form no bonds (complex 1), form a hydrogen bond to a solvent molecule (complex 2), or participate in a polymeric chain formation by binding to a Cu atom from an adjacent complex (compounds 3 and 4). The dinuclear cores in 1–3 have similar metric parameters: the Cu⋯Cu separations are 2.6845(15), 2.6955(5), and 2.6997(11) Å, respectively; the Cu–O distances in 1–3 average 1.969(11), 1.964(9), and 1.965(3) Å, whereas the Cu–N distances are longer and measure 2.1919(16), 2.1917(16), and 2.210(4) Å respectively.

Dinuclear complex 1 resides on a crystallographic inversion centre, thus only one half of it is symmetry-independent (Fig. 1). The Cu atom coordination environment is octahedral: four oxygen atoms from four bridging carboxylates form the equatorial plane, a pyrazole N atom occupies one apical position, and the other Cu atom resides at the other. The Cu atom is displaced from the equatorial plane toward the nitrogen by 0.2201(5) Å. The Cu⋯Cu separation in the complex (2.6845(15) Å) corresponds to a weak cuprophilic interaction and falls within the distance range observed for similar complexes. A Cambridge Structural Database (CSD) search for relevant structures returned the range of 2.565–2.707 Å for such interactions.

Similarly, to 1, the dinuclear compound 2·7H_2_O crystallizes with discrete complexes containing four bridging carboxylates, and two terminal pyrazoles. But unlike compound 1, there are *ca.* seven molecules of solvent water per dinuclear complex 2. These seven water molecules are distributed over eight crystallographic positions. Complex 2 resides on a crystallographic inversion centre, thus only one half of it is symmetry independent. The coordination environment of the Cu centre consists of four oxygens from four carboxylates in the equatorial plane and a N atom and the other Cu atom in the axial positions (Fig. 2a). The Cu atom is displaced by 0.2316(8) Å from the equatorial plane toward the nitrogen. The Cu⋯Cu distance of 2.6955(4) Å is statistically significantly longer than the corresponding distance in 1, and is also at the higher end of the typical range for such interactions in these compounds. The pyrazole linker has two N atoms, one from each pyrazole, that can ligate a metal centre. One of these nitrogen coordinates to the metal centre whereas the other forms a strong hydrogen bond of the type O–H⋯N (O⋯N separation = 2.663(2) Å, the O–H⋯N angle = 178(3)°) with a hydroxyl group from a neighbouring complex. These hydroxyl–pyrazole hydrogen-bonding interactions connect the complexes into two-dimensional networks parallel to the (1̄01) plane. These networks stack in such a way that there are solvent-accessible voids in the crystallographic [100] direction. These voids are populated by the solvent water molecules that form hydrogen bonds among themselves and with the complexes, thus all entities are hydrogen-bonded into a three-dimensional framework (Fig. 2b).

Complex 3 crystallizes as a merohedral twin with a 0.463(2) second twin component contribution. There is also extensive positional disorder in each ligand with an 85.04(15)% of the major component contribution. In the following discussion the major disorder component only will be considered. The pyrazolate-carboxylate coordination polymer 3 contains dimeric Cu units similar to those in 1 and 2. The dinuclear core in 3 resides on a crystallographic inversion centre and is similar in its geometry to the cores in 1 and 2·7H_2_O. Only one half of the complex is symmetry-independent. The coordination sphere of each Cu atom consists of four equatorial oxygen atoms from four carboxylates with a pyrazole N atom and second Cu atom in the apical positions (Fig. 3a). The Cu atom is displaced from the equatorial plane toward the nitrogen by 0.2356(17) Å. In contrast to 1 and 2, complex 3 is polymeric. The bidentate linker L bridges adjacent dinuclear Cu units by coordinating with one unsubstituted pyrazole.

N atom to one Cu atom and with the other unsubstituted pyrazole nitrogen to another Cu atom. These one-dimensional polymeric chains propagate in the crystallographically equivalent [110] and [1̄10] directions (Fig. 3b). The Cu⋯Cu separation of 2.6997(11) Å is the longest among 1–3 and the difference is statistically significant. There are 13 coordination polymers of this type reported to the CSD with the Cu⋯Cu cuprophilic interactions ranging between 2.565–2.691 Å thus the separation in 3 slightly exceeds this range.

In the pyrazole-carboxylate coordination polymer 4 there are two symmetry-independent Cu centres (Fig. 4a) that are bridged by the ligand L. The one-dimensional polymer propagates in the crystallographic direction [101], Fig. 4b. Each Cu atom resides on a crystallographic inversion centre, thus there is only one symmetry-independent pyrazole and benzoate coordinated to it; their symmetry-related mates complete the coordination environment about each Cu atom. In contrast to metal centres in 1–3, the Cu atoms in 4 possess distorted *trans*-square-planar environments comprised of two pyrazole nitrogens and two carboxylate oxygen atoms from two benzoate ligands. The two remaining carboxylate oxygen atoms from each benzoate form weak contacts with the Cu centres to complete their square bipyramidal coordination environment. For the rest of this paragraph we will list metric parameters averaged over the two metal centres. The average distances in the complexes are Cu–O (eq) 1.95(2) Å, Cu–O (axial) 2.65(10), Cu–N 1.9947(13) Å; the Cu–O distances between the two Cu centres are dissimilar whereas the Cu–N distances are very close in length. Apical atom O2 of one benzoate is 0.78 Å further away from atom Cu1 than its equatorial partner O1; similarly, atom O8 of the other benzoate is 0.62 Å is more remote from atom Cu2 than atom O7. The weak Cu1⋯O2 (2.7178(9) Å) and Cu2⋯O8 (2.5817(8) Å) interactions fall in the typical range observed for asymmetrically coordinated carboxylates, numerous examples of which have been structurally characterized and reported to the CSD.

### Magnetic measurements

**Fig. 5:**
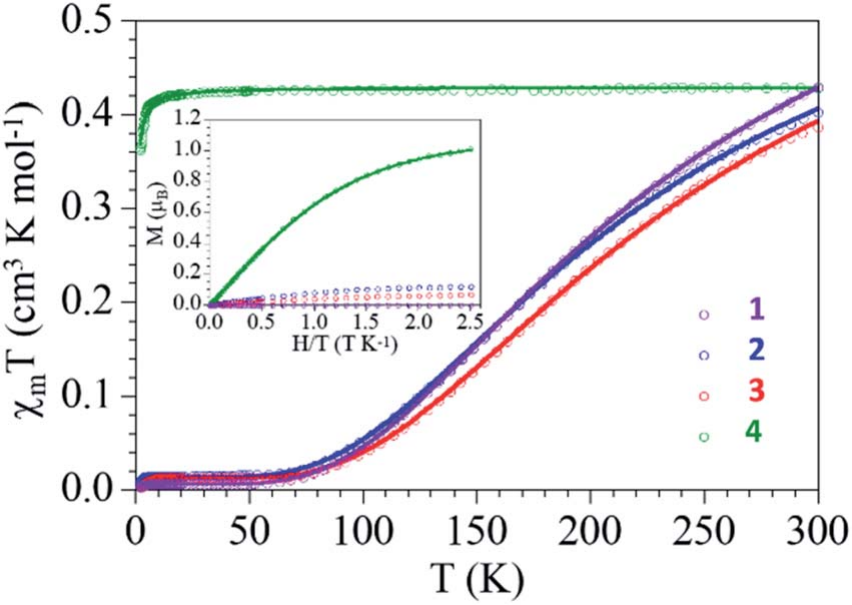
Thermal dependence of *χ*_m_*T* per Cu(ii) ion and isothermal magnetization at 2 K (inset) for compounds 1–4. Solid lines are the best fit to the models (see text).

The thermal variation of the product of the molar magnetic susceptibility per Cu(ii) ion times the temperature (*χ*_m_*T*) for complexes 1–3 show, as expected, very similar behaviors: they show at room temperature a *χ*_m_*T* value of *ca.* 0.4 cm^3^ K mol^−1^, slightly higher than the expected one for a magnetically isolated *S* = 1/2 Cu(ii) ion (0.375 cm^3^ K mol^−1^) but within the normal range observed for other Cu(ii) dimers. When the samples are cooled, *χ*_m_*T* decreases and reach a plateau with an almost zero value below *ca.* 70 K (Fig. 5).

This behaviour clearly indicates the presence of a strong antiferromagnetic Cu(ii)–Cu(ii) coupling inside the dimer and a very small paramagnetic monomeric impurity (since the *χ*_m_*T* value at low temperatures is not zero). Accordingly, we have fit the magnetic properties to a simple Bleaney–Bowers dimer model plus a *S* = 1/2 paramagnetic impurity (c).^53^ This simple model reproduces very satisfactorily the magnetic properties of compounds 1–3 in the whole temperature range with the following parameters: *g* = 2.124, *J* = −298 cm^−1^ and *c* = 0.8% for 1, *g* = 2.054, *J* = −291 cm^−1^ and *c* = 1.9% for 2 and *g* = 2.053, *J* = −320 cm^−1^ and *c* = 1.6% for 3 (solid lines in Fig. 5, the Hamiltonian is written as *H* = −*JS*_1_*S*_2_). The coupling constant values are in good agreement with reported^49,50^ and calculated^54^ values for dimeric carboxylate-bridged copper systems of general formula [Cu_2_(μ-RCOO)_4_L_2_]. For instance, the coupling constant for dimeric copper(ii) benzoate with pyridine has been reported to be −328 cm^−1^; a value that corresponds well with that for complex 3 (−320 cm^−1^).^50^ The presence of the monomeric impurity is very common and is simply due to a partial decomposition of the dimers.

Compound 4 also shows a room temperature value of *ca.* 0.42 cm^3^ K mol^−1^ (slightly higher than the spin only value for a Cu(ii) ion) but a different thermal behaviour: *χ*_m_*T* remains constant down to *ca.* 10 K, where it shows a decrease to reach a value of 0.36 cm^3^ K mol^−1^ at 2 K (Fig. 5). This behavior indicates that compound 4 behaves essentially as a paramagnet with a very weak antiferromagnetic Cu⋯Cu coupling (mediated through the L bridge). Since the structure of 4 shows the presence of Cu(ii) chains, the magnetic properties were fitted with a simple model for regular *S* = 1/2 chains.^55^ This model reproduces very satisfactorily the magnetic properties in the whole temperature range with *g* = 2.139 and an exchange parameter, *J* = −0.35 cm^−1^ (Fig. 5, the hamiltonian is written as *H* = −*JS*_*i*_*S*_*i*+1_). This low *J* value confirms the presence of a very weak antiferromagnetic interaction between the Cu(ii) ions in the chain.

The isothermal magnetization of the complexes (inset in Fig. 5) shows that compounds 1–3 are essentially diamagnetic, due to the strong antiferromagnetic coupling, and present at low temperatures a residual magnetization arising from the small monomer fraction. In contrast, compound 4 is essentially paramagnetic and reaches a saturation value close to 1.0 *μ*B, the expected one for a *S* = 1/2. In fact, the magnetization plot of compound 4 can be well reproduced with a Brillouin function for *S* = 1/2 with a *g* value of 2.116, in agreement with the result obtained in the fit of the thermal variation of *χ*_m_*T*.

### Polymerization reactions

The initiator performance of complexes 1–4 was investigated on the ROP of d,l-lactide and ε-caprolactone. The complexes showed good stability in air and were all soluble in hot toluene (above 70 °C). ε-CL polymerization was performed at 110 °C in solvent-free medium and at different [monomer] : [initiator] ratios ([M] : [I] = 50 : 1, 1500 : 1 and 3333 : 1). Polymerization of d,l-lactide was first attempted in a toluene solution at 70 °C but no opening of the ester ring was observed after 24 h. Increasing the temperature to 110 °C resulted in successful ring opening of the monomer at [M]/[I] ratio of 100 : 1. The effect of alcohol on the polymerization rate was also studied using methanol as an additive in the polymerization of d,l-lactide.

Kinetics studies were carried out for the polymerization of ε-CL and d,l-lactide using complexes 1–4 to investigate the effect of initiator structure on the polymerization reactions and polymer properties. The kinetics study was followed by ^1^H NMR spectroscopy, aliquots were taken at regular time intervals and their ^1^H NMR spectra were recorded to determine the percentage conversions (from monomer to polymer).

#### Kinetics study for ε-CL ROP

In order to study the effectiveness of all four copper complexes towards initiating the ε-CL polymerization, complexes 1–4 were used in a [M] : [I] ratio of 50 : 1 at 110 °C in a solvent-free medium. This study shows that all the reactions were complete within 48 h, except for initiator 1 (Fig. S1†). The performance of the initiators decreased in the order: 4 > 2 > 3 > 1. The best performing initiator for this polymerization is polymeric complex 4, [Cu_2_{(3,5-NO_2_)_2_-C_6_H_3_COO}_4_L_2_]_*n*_, while the slowest initiation was shown by complex 1, [Cu_2_(C_6_H_5_COO)_4_(L)_2_].

**Fig. 6:**
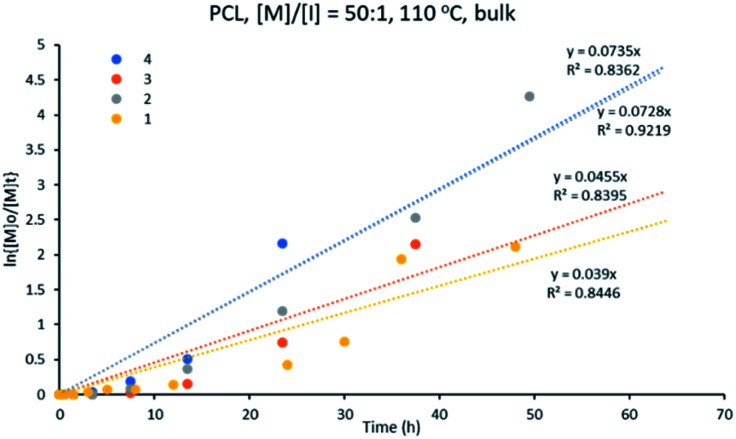
Kinetic plot for ε-CL polymerization with [M] : [I] of 50 : 1 at 110 °C, using initiators 1–4, in bulk.

Fig. 6 shows that the variation of ln{[M]_o_/[M]_t_} with time follows a linear relationship, indicating a pseudo-first order kinetics of ε-CL for the different initiator systems studied. The apparent rate constant (*k*_app_), obtained from the slope of the graph of ln{[M]_o_/[M]_t_} *versus* time, was found to vary in the range of 0.039–0.073 h^−1^ (Table 1) for the ε-CL polymerization (see eqn (1) and (2) in ESI†). These results can be compared to similar initiator systems for bis(pyrazole)copper benzoate where the rate of polymerization with the bis(pyrazole)copper benzoate initiators are much faster than observed for 1–4.^41^ Nevertheless, in both studies the best performance was shown by the nitro-benzoate derivatives, indicating that catalytic performance of initiators is influenced by the substitution on the benzoate ligands.

**Table 1:** ROP of ε-CL and d,l-lactide using complexes 1–4 at [M] : [I] = 50 : 1 for ε-CL and 100 : 1 for d,l-lactide

Entry	Complex	PCL					PLA				
		Conversion (%)	k_app_ (h^−1^)	*M*_n_ (^1^H NMR)	*M*_n_ (SEC)^a^	PDI	Conversion (%)	k_app_ (h^−1^)	*M*_n_ (^1^H NMR)	*M*_n_ (SEC)^b^	PDI
1	1	98	0.039	4065	858	2.16	97	0.048	1695	602	1.64
2	2	92	0.072	3160	—	—	100	0.053	2953	—	—
3	3	93	0.045	3900	—	—	91	0.003	1877	—	—
4	3^c^	—	—	—	—	—	91	0.030	—	—	—
5	4	100	0.073	5250	—	—	96	0.012	1812	—	—

a Using a correcting factor 0.56 for *M*_n_.

b Using a correcting factor 0.58 for *M*_n_.

c Reaction was carried out using methanol as additive.

The effect of changing the [M] : [I] ratio was studied for initiator system 4 by running the polymerization using [M] : [I] ratios of 1500 : 1 and 3333 : 1 (Fig. S2†). With a [M] : [I] ratio of 1500 : 1 we observe a significant drop in the polymerization rate from 0.073 to 0.013 h^−1^. The decrease in the number of active sites available for polymerization may account for the slower polymerization. Further lowering of the initiator concentration to [M] : [I] = 3333 : 1 only caused an insignificant change in rate (*k*_app_ = 0.012 h^−1^) compared to [M] : [I] of 1500 : 1. This may be due to steric hindrance between the growing polymer chains in the 1500 : 1 molar ratio system.

#### Kinetics study for d,l-lactide ROP

**Fig. 7:**
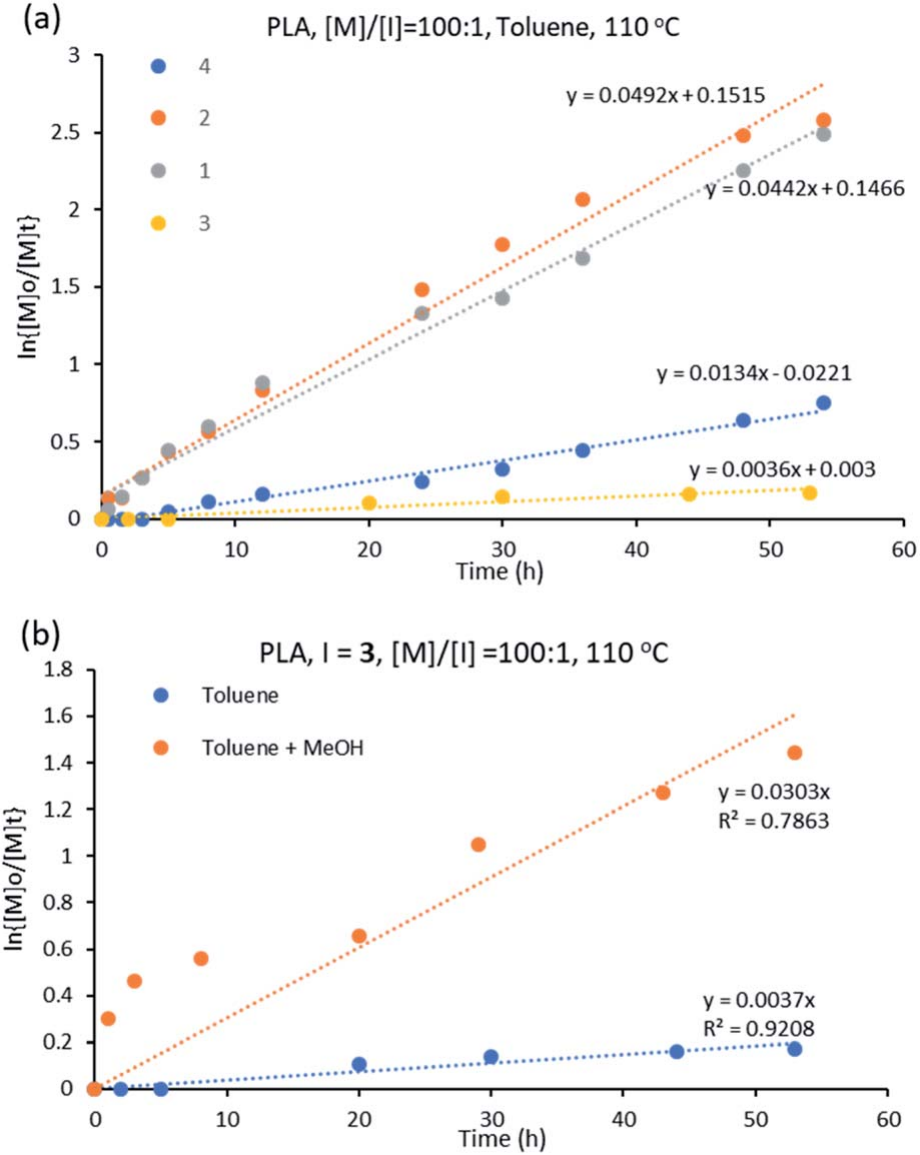
(a) Kinetic plot for d,l-lactide using initiators 1–4 in toluene at 110 °C with [M] : [I] = 100 : 1 (b) ROP of d,l-lactide using 3 at [M]/[I] = 100 : 1 and at 110 °C in different solvents.

d,l-Lactide polymerization was performed using complexes 1–4 with [M] : [I] of 100 : 1 at 110 °C in toluene. All complexes initiated the polymerization of d,l-lactide at varying rates (Fig. S3†). The initiator performance was in decreasing order of 2 > 1 > 4 > 3, showing that for the ε-CL polymerization, the trend in initiator activity is different than for the ROP of d,l-lactide for L-based complexes. The best performing initiator is 2 while the least activity is showed by complex 3 (Fig. 7a).

The constant rates for d,l-lactide ROP using initiators 1–4 varied from 0.003 to 0.053 h^−1^ (Table 1), indicating better catalytic performance of the initiators in the ROP of ε-CL than of d,l-lactide, which is in agreement with literature.^38^

Cyclic ester polymerization using [Sn(Oct)_2_] shows optimum performance when butanol is present in the reaction.^56^ In this respect, the polymerization of d,l-lactide was repeated for complex 3 with methanol as co-initiator (with a volume ratio of toluene : methanol = 50 : 1). The polymerization rate increased when methanol was added, with the rate constant increasing from 0.003 to 0.030 h^−1^, values extracted from the plot of ln{[M]_o_/[M]_t_} *versus* time (Fig. 7b). The influence of the alcohol in the polymerization process agrees with the literature.^57^

Although we have not studied the mechanism of the ROP for initiators 1–4, given their similarity with the [Al(O^*i*^Pr)_3_] system,^58^ both with M–O bonds, the ROP mechanism in 1–4 is expected to occur *via* coordination-insertion.

### Characterization of polymers

#### Polycaprolactone (PCL) characterization

The molecular weights of the PCL produced were first determined by ^1^H NMR spectroscopy (using eqn (4) in ESI†) and by size exclusion chromatography (SEC) in the case of 1. The results obtained are summarized in Table 1. The highest *M*_n_(NMR) was obtained for complex 4 (5250 Da), while 2 gave PCL of lowest molecular weight (3160 Da). SEC was run on the PCL synthesized with 1 and using THF (HPLC grade) as eluent. The *M*_n_(SEC) value and the polydispersity index, (PDI = *M*_w_/*M*_n_), are given in Table 1 (entry 1). SEC molecular weights, calibrated on polystyrene standards were multiplied by 0.56 as a correcting factor for PCL.^59^ The molecular weight of PCL obtained from initiator 1 measured by SEC is very small (858 Da) compared to those of PCL synthesized using [Al(O^*i*^Pr)_3_] and [Sn(Oct)_2_] as initiators, with molecular weights of 41 800 and 5700 Da, respectively.^60^ This may be explained in terms of accessibility of the metal centre since mononuclear [Al(O^*i*^Pr)_3_] and [Sn(Oct)_2_] have less bulky ligands and less encumbered metal centres than paddle-wheel copper benzoates and, therefore, show a better monomer-metal interaction than in complexes 1–4.

#### Poly(d,l-lactide) (PLA) characterization

d,l-Lactide polymerization and the PLA obtained by using complexes 1–4 were characterized in a similar manner as PCL. The *M*_n_(NMR) values for PLA synthesized in toluene are summarized in Table 1. The highest *M*_n_(NMR) is obtained for initiator system 2 (2953 Da), while the lowest is for initiator 1 (1695 Da). Furthermore, the PLA synthesized by adding methanol as co-initiator have *M*_n_(NMR) comparable with the PLA prepared without methanol. Molecular weight from SEC, (corrected by a correcting factor of 0.58) obtained for PLA with initiator 1 is much lower (601 Da).

In general, two reasons account for the low molecular weight of the polymers: inefficient catalyst and *trans*-esterification reactions.^61,62^ The latter can either be inter- or intramolecular, leading to lower molecular weight and/or broader PDI. Hence, the lower molecular weights and wider PDI of polymers derived from complexes 1–4 in comparison to their mononuclear analogues indicates that the paddle-wheel structures may be the cause for the drop in the initiator performance. Moreover, the benzoate bridging ligands together with the rigidity of the structure and the bulkiness around the metal centre, hinders the polymer chain growth resulting in short chains, low molecular weight and lower catalytic activity for 1–4. This idea is supported by the better performance of similar copper paddle wheel structures bearing acetate bridging carboxylates, as reported by Ojwach and co-workers.^38^

The SEC of most PLA and PCL show more than one peak: a main peak and other much smaller peaks. The latter are attributed to the formation of cyclic structures, a result of intramolecular transesterification reactions, involving backbiting. Comparing these two polymers, we can observed: (i) a more controlled polymerization for lactide ROP than for ε-CL, as depicted by the narrower molecular weight distribution, (measured as the polydispersity index, PDI) and (ii) a lesser intermolecular transesterification for PLA (PDI = 1.64) *versus* PCL (PDI = 2.16). In order to study the polymer structure of the produced PCL and PLA with initiators 1–4, we have used MALDI-ToF mass spectrometry.

#### MALDI-ToF MS of PCL and PLA

Ma and Okuda^63^ and Phomphrai *et al.*^64^ have studied the MALDI-ToF mass spectra of PLA and PC, respectively, as a means of finding the types of polymer chains formed. Three types of polymers have been reported from MALDI ToF mass spectrometry: namely type A, where the polymer chain has –OH ending group as a result of workup process; type B, where the ligand from the initiator is still attached to the polymer chain and type C, formed by cyclic oligomers or polymers (Fig. S4†).

**Fig. 8:**
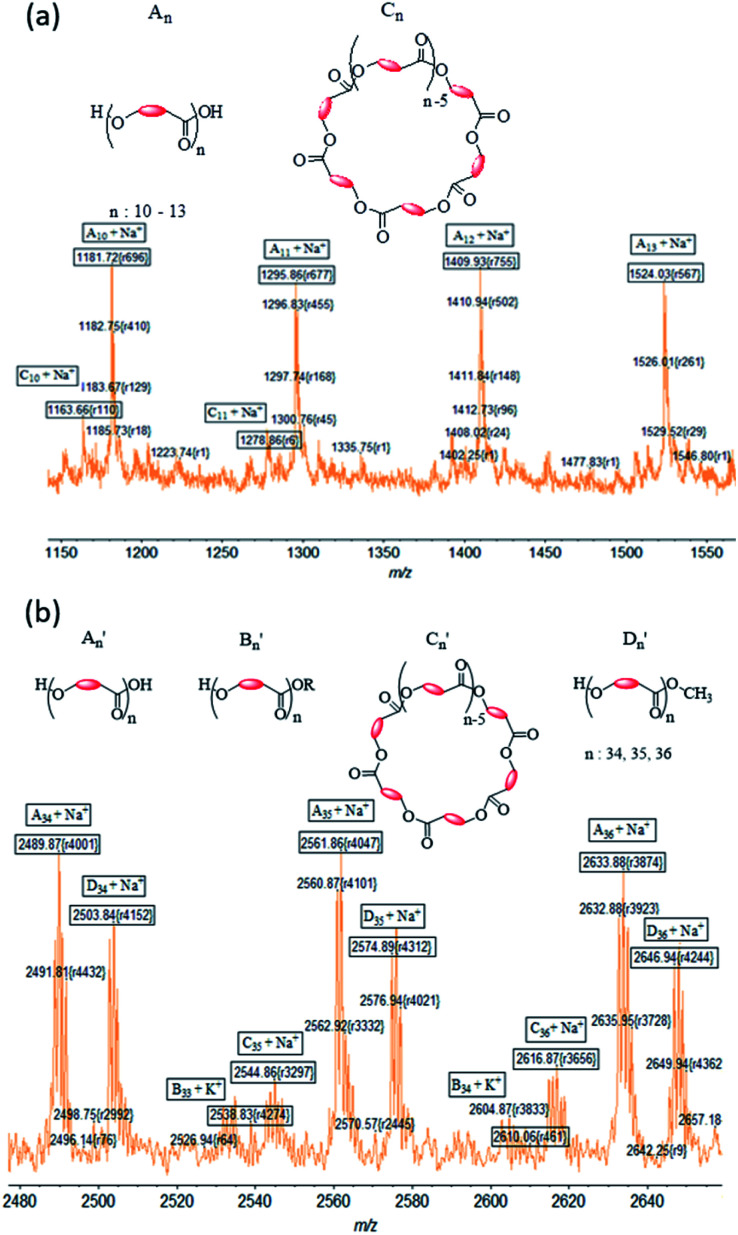
Expansion of MALDI-TOF mass spectrum of (a) PCL in the region *m*/*z* = 1150–1550 and (b) PLA in the region *m*/*z* = 2480–2660 made using 1.

The MALDI ToF spectrum of PCL synthesized using complex 1 shows an envelope-shaped spectrum (Fig. S5a†).^65^ All peaks are associated with either Na^+^ or K^+^ ion from the matrix. When the region *m*/*z* = 1150–1550 is expanded, a pattern is revealed that repeats itself several times (Fig. 8a). The most intense peaks correspond to a polymer chain that has hydroxyl end groups (type A). Peaks associated to cyclic PCL of type C were also observed; but type B PCL were not identified in the system, indicating that the polymer chains hydrolyzed during the workup process.

Molecular weights (*m*/*z* values) for PLA obtained with initiator 1 (Fig. S5b†) are much higher than those for PCL. An expansion of the PLA spectrum reveals the presence of more peaks in the repeat segment of the spectrum than was observed for PCL (Fig. 8). Using Na^+^ in calculating *m*/*z* results in a good correlation between the found and calculated values for the most intense peaks. These peaks are assigned to type A′ PLA, whereas lower intensity peaks are assigned to type C′ PLA. Type B′ PLA were also identified in the spectrum when the doping ion is considered to be K^+^. Surprisingly we identified an additional set of peaks (Fig. 8b), separated by 72 Da, which we assigned to a new polymer described as type D′ PLA that has a methoxy end group. PLA with –OCH_3_ groups are likely to be formed during the quenching of the reaction with methanolic–HCl solution.

Table 2 is a summary of the types of PCL and PLA that were produced with all four initiators and shows the significant role that an initiator plays in the polymerization reaction. It is clear from the intensity peaks that type A polymer with hydroxyl end groups, resulting from breaking of the metal–polymer bond, is the predominant polymer type. The same analysis shows that there is a very low amount of type B polymer that bears the initiator ligand as end group. This result indicates that the process leading to the ligand at the end of the polymerization process is not significant in the reaction.

**Table 2:** Peak assignments for PCL and PLA derived from 1–4

I	PCL			PLA		
	*m*/*z* (obs)	*m*/*z* (calc)	Assignment	*m*/*z* (obs)	*m*/*z* (calc)	Assignment
1	1163	1164	C_10_ + Na^+^	2489	2489	A′_34_ + Na^+^
1	1181	1182	A_10_ + Na^+^	2503	2503	D′_34_ + Na^+^
1	1278	1278	C_11_ + Na^+^	2538	2537	B′_33_ + K^+^
1	1295	1286	A_11_ + Na^+^	2544	2543	C′_35_ + Na^+^
2	1867	1867	A_16_ + Na^+^	1351	1353	A′_18_ + K^+^
2	1981	1981	A_17_ + Na^+^	1390	1391	A′_19_ + Na^+^
3	953	954	A_8_ + Na^+^	1422	1423	D′_19_ + Na^+^
3	995	992	B_7_ + K^+^	1464	1463	C′_20_ + Na^+^
4	3008	3008	A_26_ + Na^+^	2129	2129	A′_29_ + Na^+^
4	3034	3038	D_26_ + K^+^	2143	2143	D′_29_ + Na^+^
4	3122	3122	A_27_ + Na^+^	2184	2183	C′_30_ + Na^+^

## Conclusions

Four pyrazolyl copper(ii) benzoates have been synthesized *via* one-pot reactions and while the pyrazolyl ligand does not influence the structure of the complexes formed, the ancillary benzoate ligands do. Three of the copper complexes (1–3) with less bulky benzoate ligands (4-OH-C_6_H_4_COO^−^, 2-Cl-C_6_H_4_COO^−^ and C_6_H_5_COO^−^) have paddle-wheel bimetallic structures, whereas the complex formed by the bulky benzoate (3,5-NO_2_)_2_-C_6_H_3_COO^−^ ligand, 4, does not. The different structural types give rise to very different magnetic behaviours. Complex 4, that presents a linear Cu(ii) chain, behaves as an antiferromagnetically coupled *S* = 1/2 regular chain with a very weak antiferromagnetic coupling (*J* = −0.35 cm^−1^). In contrast, complexes 1–3, that present Cu(ii) paddle wheel dimeric structure with four carboxylate bridging ligands, behave as antiferromagnetic *S* = 1/2 dimers with strong antiferromagnetic coupling constants (*J* = −291, −320 and −298 cm^−1^ in 1–3, respectively). All four complexes are able to initiate ring opening polymerization of both ε-caprolactone and d,l-lactide at high temperatures. The ability of these complexes in initiating the polymerization reaction strongly depends on the structure of the complexes. Polymeric initiator 4 exhibits the best performance in the ROP of ε-CL, while initiator 2 is the best initiator for d,l-lactide ROP. All the copper complexes produced polymer of low molecular weight as determined by NMR spectroscopy and SEC. MALDI-ToF MS shows that the type A polymers, bearing hydroxy end groups, are the major products. Transesterification leading to cyclization of the polymer chains may account for the low molecular weight and broad PDI, suggesting that paddle-wheel copper benzoates may not be the preferred type of structure to obtain monodispersed polymers of high molecular weight.

## Experimental

### General procedures

Syntheses were performed under a dry nitrogen atmosphere using standard Schlenk techniques. All solvents were of analytical grade and were dried and distilled prior to use. Toluene and dichloromethane were dried and distilled from sodium/benzophenone and P_2_O_5_ respectively. [Cu(OAc)_2_], C_6_H_5_COOH, 4-OH-C_6_H_4_COOH, (3,5-NO_2_)_2_-C_6_H_3_COOH, 2-Cl-C_6_H_4_COOH, magnesium sulphate, tetrabutylammonium bromide and ε-caprolactone (98%) were obtained from Sigma-Aldrich and used as received. d,l-Lactide was obtained from Purac biochem and used as received. The ligand L was synthesized following literature procedure.^66^

The NMR spectra were recorded in chloroform-d (CDCl_3_) on an Oxford Gemini 2000 instrument (300 MHz for ^1^H NMR and 75 MHz for ^13^C{^1^H} NMR) and a Bruker Ultrashield 400 instrument (400 MHz for ^1^H NMR and 100 MHz for ^13^C{^1^H} NMR) at room temperature. ^1^H and ^13^C{^1^H} NMR chemical shifts were referenced to the residual signals of the protons or carbons of the NMR solvents and are quoted in *δ* (ppm): CDCl_3_ at 7.24 and 77.0 ppm for ^1^H and ^13^C{^1^H} NMR spectra, respectively. Infrared spectra were recorded on a Bruker FR-IR Tensor27 spectrometer fitted with an ATP-IR probe. Elemental analyses were performed on a Vario Elementar microcube CHNS analyzer at Rhodes University, South Africa. ESI-MS spectra were recorded on a Waters API Quattro Micro spectrophotometry at the University of Stellenbosch, South Africa. Magnetic susceptibility measurements were performed with an applied magnetic field of 0.1 Tesla in the temperature range 2–300 K on polycrystalline samples of compounds 1–4 (with masses of 36.57, 38.30, 32.95 and 34.94 mg, respectively) with a Quantum Design MPMS-XL-5 SQUID susceptometer. The isothermal magnetization measurements were performed on the same samples at 2 K with fields from −5 to 5 Tesla. Susceptibility data were corrected for the sample holder and the diamagnetic contribution of the salts using Pascal's constants.^66^ Molecular weight and molecular weight polydispersity of polymers were determined by size exclusion chromatography (SEC) and by Matrix-Assisted Laser Desorption/Ionization Time-of-Flight mass spectrometry (MALDI-ToF MS). SEC analysis was performed at the University of Mauritius using a WGE Dr Bures Q1000 Gel Permeation Chromatogram and at Kyoto University, Japan, using a JASCO GULLIVER system (PU-980, CO-965, RI-930, and UV-1570) equipped with polystyrene gel columns (Shodex columns K803, K804, K805), using THF as an eluent at a flow rate of 1.0 mL min^−1^, calibrated by polystyrene standards at 40 °C. MALDI-ToF MS measurements were made at Kyoto University and University of Tokyo, Japan using a Shimadzu Biotech Axima CFRplus. Thermal analysis of the polymers was carried out using a Mettler-Toledo DSC 822^e^ and Perkin Elmer STA 6000 Simultaneous Thermal Analyzer.

### X-ray crystallography

Crystal evaluation and data collection were performed on a Bruker Quazar SMART APEXII diffractometer with Cu for 3 and Mo for all the other complexes (1, 2 and 4). The crystals were attached to the tip of a MiTeGen MicroMount©. Each crystal was mounted in a stream of cold nitrogen at 100(1) K and centered in the X-ray beam by using a video camera. The initial cell constants were obtained from three series of ω scans at different starting angles. The reflections were successfully indexed by an automated indexing routine built in the APEXII program suite. The absorption correction was based on fitting a function to the empirical transmission surface as sampled by multiple equivalent measurements.^67,68^ A successful solution by the direct methods provided most non-hydrogen atoms from the E-map. The remaining non-hydrogen atoms were located in an alternating series of least-squares cycles and difference Fourier maps. All non-hydrogen atoms were refined with anisotropic displacement coefficients. All hydrogen atoms were included in the structure factor calculation at idealized positions and were allowed to ride on the neighbouring atoms with relative isotropic displacement coefficients. CCDC 2051015–2051018 contain the supplementary crystal data for complexes 1–4.

### Synthesis of the copper complexes 1–4

Two methods were used for the complexation reactions: method A and method B.

Method A involves a one pot, overnight reaction between copper(ii) acetate, benzoic acid and the pyrazolyl ligand, L in a 1 : 2 : 1 mole ratio in methanol (50 mL). Copper(ii) acetate and benzoic acid were first refluxed in methanol for 5 h and a solution of L in methanol (2 mL) is then added dropwise with constant stirring and heating. After 16 h of refluxing, the solution is allowed to cool to room temperature and slow evaporation of the solvent results in the formation of crystals. The copper products were characterized by FTIR, elemental analysis, mass spectrometry and magnetic measurements. Complexes 2–4 were synthesized by this method.

Method B is an adaptation of the procedure used by Baruah^69^ to synthesize metal carboxylate complexes with pyrazoles. The method of preparation is a one pot, room temperature reaction between copper(ii) acetate, benzoic acid and L in the same mole ratio as method A. Copper(ii) acetate is stirred with benzoic acid in methanol (15 mL) at room temperature for 30 min and then a solution of L in toluene (5 mL) is added to the mixture and stirred for a further 30 min. The product of the reaction is isolated by evaporation of the solvent. Complex 1 was prepared using method B.

#### [Cu_2_(C_6_H_5_COO)_4_(L)_2_] (1)

Complex 1 was prepared following method B, using [Cu(OAc)_2_] (0.20 g, 1.00 mmol), C_6_H_5_COOH (0.24 g, 2.00 mmol) and L (0.29 g, 1.00 mmol) in a toluene : methanol mixture (50 mL of 1 : 1 volume ratio). During the reaction, a green solid precipitated from the reaction mixture. Compound 1 was then isolated by filtration and dried under vacuum. The solid was re-dissolved in DMSO to obtain green single crystals upon slow evaporation of the solvent. Yield: 0.34 g (57%). FTIR: 1627 cm^−1^ (*ν*_C=O_). *μ*_eff_: 1.938 BM. Anal. Calc. for C_46_H_42_Cu_2_N_4_O_8_·CH_3_OH: C, 60.18%; H, 4.94% and N, 5.97%. Found: C, 59.20%; H, 4.709% and N, 6.08%.

#### [Cu_2_(4-OH-C_6_H_4_COO)_4_L_2_] (2)

[Cu(OAc)_2_] (0.20 g, 1.00 mmol), 4-OH-C_6_H_4_COOH (0.28 g, 2.00 mmol) and L (0.29 g, 1.00 mmol) in methanol (50 mL) were reacted following method A. Complex 2 was obtained as green solid upon evaporation of methanol under vacuo. Green crystals, suitable for X-ray analysis, were obtained by slow evaporation of a CH_2_Cl_2_ solution of 2. Yield: 0.43 g (68%). ES-MS: *m*/*z* (%); 494 [M-4-OH-C_6_H_4_COO]^+^, 100. FTIR: 1625 cm^−1^ (*ν*_C=O_). *μ*_eff_: 1.897 BM. Anal. Calc. for C_32_H_32_CuN_4_O_6_·0.75CH_2_Cl_2_: C, 56.53%; H, 4.85% and N, 8.05%. Found: C, 56.98%; H, 5.36% and N, 7.56%.

#### [Cu_2_(2-Cl-C_6_H_4_COO)_4_L_2_] (3)

Complex 3 was synthesized using [Cu(OAc)_2_] (0.20 g, 1.00 mmol), 2-Cl-C_6_H_4_COOH (0.31 g, 2.00 mmol) and L (0.29 g, 1.00 mmol) in methanol (50 mL), which afforded green crystals by slow evaporation of the solvent. Yield: 0.44 g (66%). FTIR: 1631 cm^−1^ (*ν*_C=O_). *μ*_eff_: 1.810 BM. ES-MS: *m*/*z* (%); 692 [M + Na]^+^, 20; 512 [M-2-Cl-C_6_H_4_COO]^+^, 100. Anal. Calc. for C_32_H_30_Cl_2_CuN_4_O_4_·0.5C_7_H_8_: C, 59.62%; H, 4.79% and N, 7.83%. Found: C, 59.72%; H, 4.65% and N, 8.07%.

#### [Cu{(3,5-NO_2_)_2_-C_6_H_3_COO}_2_L]_*n*_ (4)

Complex 4 was obtained by reacting [Cu(OAc)_2_] (0.20 g, 1.00 mmol), (3,5-NO_2_)_2_-C_6_H_3_COOH (0.43 g, 2.00 mmol) and L (0.29 g, 1.00 mmol) in methanol (50 mL). A purple precipitate was observed in the reaction mixture and was filtered. The crude compound was dissolved in DMSO and single crystals were obtained upon slow evaporation of the solvent. Yield: 0.47 g (78%). *μ*_eff_: 1.854 BM. ES-MS: *m*/*z* (%); 568 [M-(3,5-NO_2_)_2_-C_6_H_3_COO]^+^, 18. FTIR: 1624 cm^−1^ (*ν*_C=O_). Anal. Calc. for C_32_H_28_CuN_8_O_12_·CH_3_OH: C, 48.80%; H, 3.97%; N, 13.80%. Found: C, 48.64%; H, 3.37%; N, 14.02%.

### Polymerization study

The catalytic activities of 1–4 towards ε-caprolactone (ε-CL) and d,l-lactide polymerizations have been evaluated under different conditions. Polymerization of ε-caprolactone was carried out in bulk using a [monomer] : [initiator] ([M] : [I]) ratio of 50 : 1 and ratios 1500 : 1 and 3333 : 1 in some cases. For the polymerization of d,l-lactide, the reaction was performed in toluene using a [M] : [I] ratio of 100 : 1. The polymerization reactions were performed in a carousel reaction station, fitted with 12 tubes, a gas distribution system and a reflux unit.

#### Synthesis of polycaprolactone

ε-CL (1.14 g, 0.01 mol) and the required amount of initiator, depending on the [M] : [I] ratio used, were weighed in a reactor tube and stirred at 110 °C. The polymerization of ε-CL was carried out in bulk with [M] : [I] ratios of 50 : 1, 1500 : 1 and 3333 : 1. After the required reaction time, the reaction mixture was quenched by rapid cooling to room temperature and the crude product was analyzed by ^1^H NMR spectroscopy in CDCl_3_. The polymers were cleaned by first dissolving the crude product in CH_2_Cl_2_, followed by the addition of cold methanol. The white precipitate formed was filtered and dried *in vacuo*.

#### Synthesis of polylactide

d,l-Lactide (1.44 g, 0.01 mol) and the required amount of initiator to keep the [M] : [I] ratio at 100 : 1 were dissolved in toluene (2.5 mL) in a reactor tube and stirred at 110 °C. After specified time intervals, each reaction was quenched by rapid cooling to room temperature. The solvent was removed *in vacuo* and the percentage conversion was determined from the ^1^H NMR spectrum. The polymer was cleaned by first dissolving the crude product in dichloromethane (15 mL) and adding a hydrochloric acid solution in methanol (0.5 M, 15 mL) to the mixture. The solution was shaken vigorously and allowed to stand to allow the layers to separate. The aqueous layer was then washed with dichloromethane (3 × 10 mL). The combined organic layer was then washed with distilled water (15 mL), followed by sodium hydroxide solution (1.0 M, 15 mL). The basic aqueous layer was washed with CH_2_Cl_2_ (3 × 10 mL). The organic phase was dried over anhydrous magnesium sulphate, filtered and concentrated *in vacuo*, yielding a white fluffy solid. In some cases, the d,l-lactide polymerization was repeated using methanol (0.05 mL) as an additive to the reaction.

## Conflicts of interest

There are no conflicts to declare.

## Supplementary Material

RA-011-D1RA00339A-s001

RA-011-D1RA00339A-s002
