# Supplementary material for: Ring opening polymerization of d,l-lactide and ε-caprolactone catalysed by (pyrazol-1-yl)copper(ii) carboxylate complexes
Source: RSC Adv. 2021 Apr 12;11(22):13475–85. doi: 10.1039/d1ra00339a (PMC8697578; doi:10.1039/d1ra00339a)
Supplement: RA-011-D1RA00339A-s001 [file RA-011-D1RA00339A-s001.pdf]

Electronic Supplementary Material (ESI) for RSC Advances.
This journal is © The Royal Society of Chemistry 2021

Ring Opening Polymerization of D,L-lactide and ε-caprolactone catalysed by (pyrazol-1-yl) copper(II) carboxylate complexes

Divambal Appavoo,[a] Lara C. Spencer,[b] Ilia A. Guzei,[b] Carlos J. Gómez-García,[c] Juanita van Wyk[a] and James Darkwa*[a]

[a] *Department of Chemical Sciences, University of Johannesburg, P.O. Box X524, Auckland Park, 2006, South Africa*

[b] *Department of Chemistry, University of Wisconsin-Madison, Madison, WI 53706 , USA*

[c] *Instituto de Ciencia Molecular (ICMol), Parque Científico de la Universidad de Valencia, 46980 Paterna, Valencia, Spain.*

Supporting Information

The crystal structures of **1**–**4** were established by standard crystallographic techniques. A typical procedure is presented for **1**. All non-routine procedures are mentioned for **1**–**4** where appropriate.

Data Collection

A green crystal with approximate dimensions 0.18 x 0.10 x 0.08 mm^3 was selected under oil under ambient conditions and attached to the tip of a MiTeGen MicroMount©. The crystal was mounted in a stream of cold nitrogen at 100(1) K and centered in the X-ray beam by using a video camera.

The crystal evaluation and data collection were performed on a Bruker Quazar SMART APEXII diffractometer with Mo Kα (λ = 0.71073 Å) radiation and the diffractometer to crystal distance of 4.95 cm.

The initial cell constants were obtained from three series of ω scans at different starting angles. Each series consisted of 12 frames collected at intervals of 0.5º in a 6º range about ω with the exposure time of 10 seconds per frame. The reflections were successfully indexed by an automated indexing routine built in the APEXII program suite. The final cell constants were calculated from a set of 9889 strong reflections from the actual data collection.

The data were collected by using the full sphere data collection routine to survey the reciprocal space to the extent of a full sphere to a resolution of 0.70 Å. A total of 24268 data were harvested by collecting 4 sets of frames with 0.4º scans in ω and φ with exposure times of 25 sec per frame. These highly redundant datasets were corrected for Lorentz and polarization effects. The absorption correction was based on fitting a function to the empirical transmission surface as sampled by multiple equivalent measurements. [1]

Structure Solution and Refinement

The systematic absences in the diffraction data were consistent for the space groups P$\bar{1}$ and P1. The centrosymmetric space group P$\bar{1}$ yielded chemically reasonable and computationally stable results of refinement [2-8].

A successful solution by the direct methods provided most non-hydrogen atoms from the E-map. The remaining non-hydrogen atoms were located in an alternating series of least-squares cycles and difference Fourier maps. All non-hydrogen atoms were refined with anisotropic displacement coefficients. All hydrogen atoms were included in the structure factor calculation at idealized positions and were allowed to ride on the neighbouring atoms with relative isotropic displacement coefficients.

The dinuclear complex resides on a crystallographic inversion centre.

The final least-squares refinement of 374 parameters against 8782 data resulted in residuals R (based on F^2 for I≥2σ) and wR (based on F^2 for all data) of 0.0300 and 0.0773, respectively. The final difference Fourier map was featureless.

In the case of **2**·7H_2O the solvent water molecules were refined with restrained geometries.

In case of **3** the crystal chosen for the data collection proved to be a merohedral twin with a 0.463(2) second twin component contribution. There is also extensive positional disorder in each ligand with a 85.04(15) % of the major component contribution. Only atoms Cu1, C10, C17 and O1–O4 are ordered. The disordered parts were refined with restraints and constraints.

Table S1 Crystallographic experimental details for **1–4**.

	**1**	**2**·7H_2O	**3**	**4**
Crystal data				
Chemical formula	$C_{64}H_{64}Cu_2N_8O_8$	$C_{64}H_{64}Cu_2N_8O_{12}$·7.074($H_2O$)	$C_{46}H_{38}Cl_4Cu_2N_4O_8$	$C_{32}H_{28}CuN_8O_{12}$
Crystal system, space group	Triclinic, *P*$\bar{1}$	Monoclinic, *P*2$_1$/*n*	Tetragonal, *P*4$_2$/*n*	Monoclinic, *P*2$_1$/*c*
Temperature (K)	100	100	100	100
a, *b*, *c* (Å)	10.468 (5), 10.853 (6), 13.430 (4)	9.7157 (3), 21.1150 (6), 17.2695 (5)	16.2772 (2), 16.2772 (2), 17.3126 (2)	14.1819 (5), 19.8388 (7), 11.9610 (5)
α, β, γ (°)	79.52 (5), 73.95 (4), 88.13 (4)	90, 101.485 (2), 90	90, 90, 90	90, 96.814 (2), 90
V (Å^3)	1441.6 (12)	3471.85 (18)	4586.93 (12)	3341.5 (2)
Z	1	2	4	4
Radiation type	Mo *K*α	Mo *K*α	Cu *K*α	Mo *K*α
μ (mm^{-1})	0.80	0.69	3.77	0.73

Crystal size (mm)	0.18 × 0.1 × 0.08	0.11 × 0.1 × 0.08	0.31 × 0.27 × 0.13	0.21 × 0.1 × 0.08
Data collection				
Diffractometer	Bruker *SMART APEX2* area detector	Bruker *SMART APEX2* area detector	Bruker *SMART APEX* II	Bruker *SMART APEX2* area detector
Absorption correction	Analytical *SADABS* (Bruker-AXS, 2007)	Analytical *SADABS* (Bruker-AXS, 2007)	Multi-scan *SADABS2016*/2 (Krause et al., 2015) was used for absorption correction.	Analytical *SADABS* (Bruker-AXS, 2007)
T_{min}, T_{max}	0.869, 0.939	0.928, 0.947	0.388, 0.640	0.862, 0.944
No. of measured, independent and observed [$I > 2\sigma(I)$] reflections	24410, 8782, 7813	57515, 10630, 8216	73846, 4450, 4331	125425, 10183, 8296
R_{int}	0.021	0.051	0.037	0.071
$(\sin \theta/\lambda)_{max}$ (Å^{-1})	0.715	0.716	0.616	0.716
Refinement				
$R[F^2 > 2\sigma(F^2)]$, $wR(F^2)$, S	0.031, 0.084, 1.05	0.046, 0.130, 1.06	0.065, 0.175, 1.09	0.030, 0.088, 1.04
No. of reflections	8782	10630	4450	10183
No. of parameters	374	460	322	485
$\Delta\rho_{max}$, $\Delta\rho_{min}$ (e Å^{-3})	0.54, -0.34	0.74, -0.47	1.67, -0.79	0.50, -0.54

Equations:

$$〖\mu\_eff= (3k/(N\beta^{\wedge}2\ )\ \chi T)〗^{\wedge}(1/2)\ \approx 2.828(\chi T)^{\wedge}(1/2) \qquad \textit{Eqn 1}$$

where k is Boltzmann constant, N is Avogadro's number, β is the Bohr magneton, χ is susceptibility and T is temperature.

$$-\frac{d[M]}{dt} = k_p[M][I] \qquad Eqn\ 2$$

where k_p is the rate constant, t is time and [M] and [I] are the monomer and initiator concentrations, respectively.

$$\ln\frac{[M]_o}{[M]_t} = k_{app}t \qquad Eqn\ 3$$

where k_{app} is k_p[I], $[M]_o$ and $[M]_t$ are the monomer concentrations at time 0 and t, respectively.

$$M_n(NMR) = Mr_{monomer} \times \frac{I_{CH}}{I_{(CH)EG}} + Mr_{initiator} \qquad Eqn\ 4$$

where M_n(NMR) is the average number molecular weight calculated from NMR, Mr is molecular weight and I_{CH} and $I_{(CH)EG}$ are the intensities of CH peaks of polymer and end group, respectively.

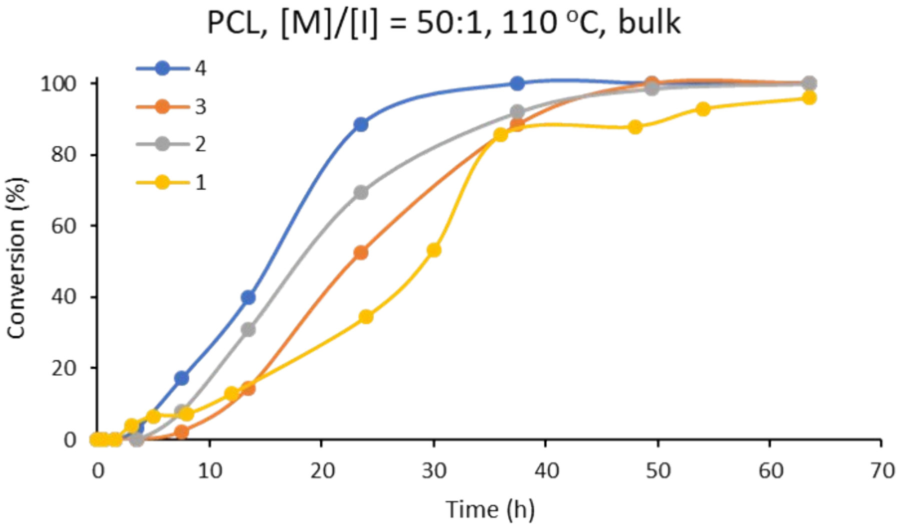

Figure S1. ROP of ε-CL using initiators **1-4** at [M]/[I] = 50:1 at 110 °C in bulk

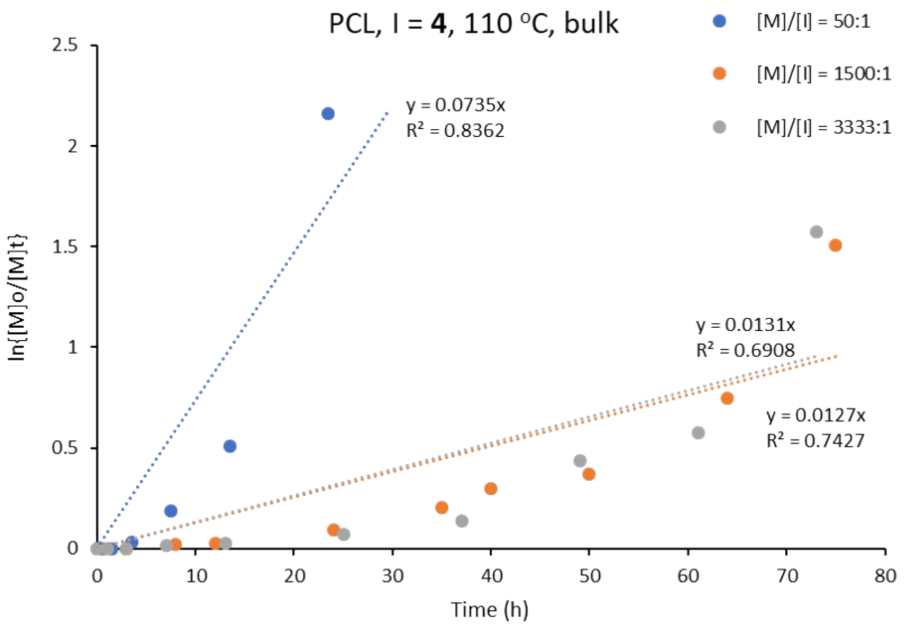

Figure S2. Effect of changing the [M]/[I] ratio on the ROP of ε-CL at 110 °C in bulk

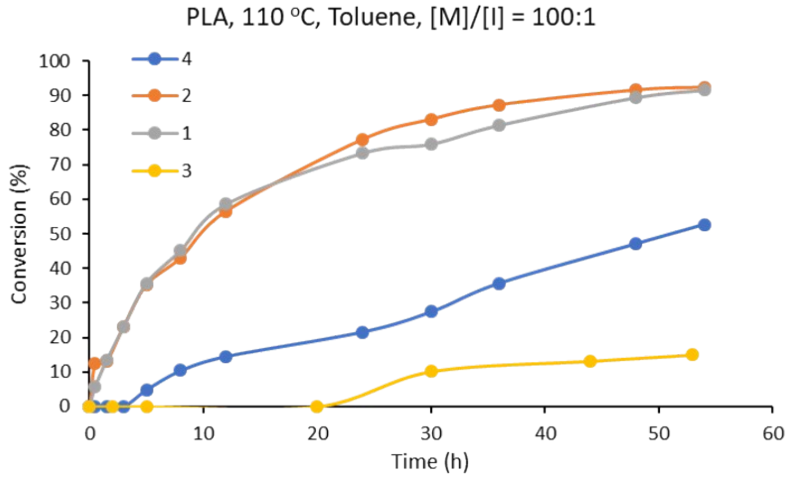

Figure S3. ROP of D,L-lactide using initiators **1-4** at [M]/[I] = 100:1, at 110 °C in toluene

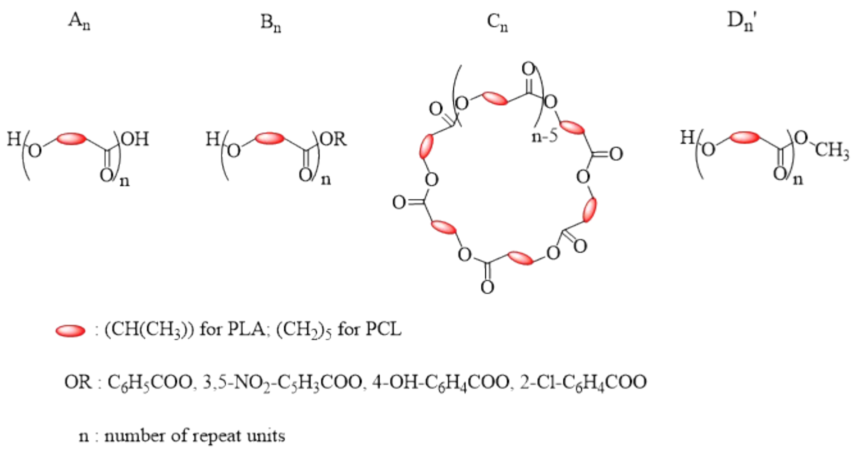

Figure S4. Structures of the different types of polymers obtained from MALDI-TOF MS.

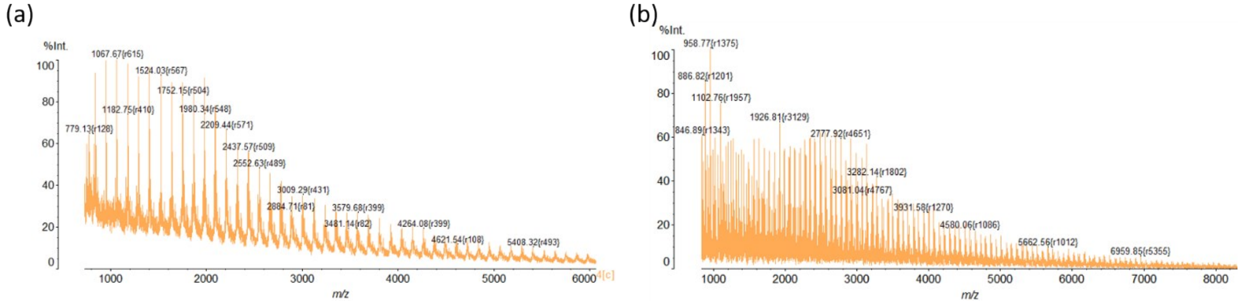

Figure S5. MALDI-ToF mass spectra of (a) PCL and (b) PLA synthesized using **1**.

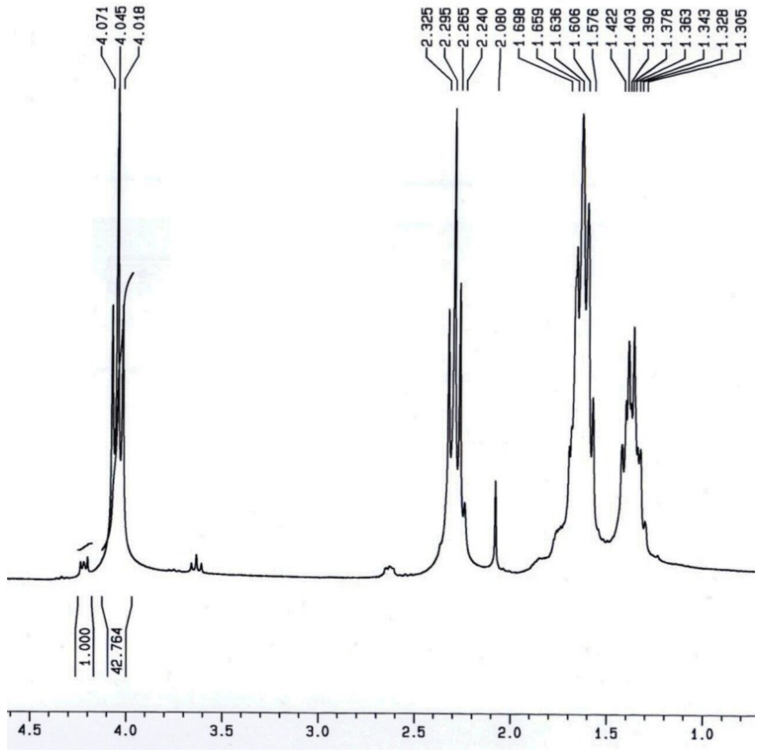

Figure S6. ^{1}H NMR spectrum a sample of PCL after workup.

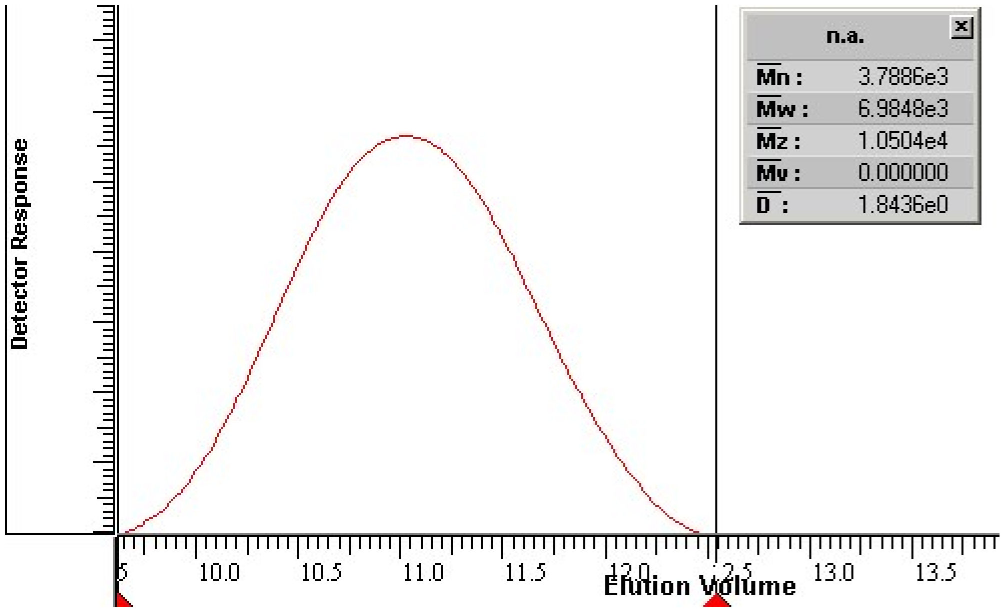

Figure S7. A size exclusion chromatogram a sample of PCL after workup.

References

1. Bruker-AXS (2018). *APEX3.* Version 2018.1-0. Madison, Wisconsin, USA.
2. Krause, L., Herbst-Irmer, R., Sheldrick, G. M. & Stalke, D. (2015). *J. Appl. Cryst.* 48. 3-10.
3. Sheldrick, G. M. (2013b). *XPREP*. Version 2013/1. Georg-August-Universität Göttingen, Göttingen, Germany.
4. Sheldrick, G. M. (2013a). The *SHELX* homepage, http://shelx.uni-ac.gwdg.de/SHELX/.
5. Sheldrick, G. M. (2015a). *Acta Cryst. A*, 71, 3-8.
6. Sheldrick, G. M. (2015b). *Acta Cryst. C*, 71, 3-8.
7. Dolomanov, O. V., Bourhis, L. J., Gildea, R. J., Howard, J. A. K. & Puschmann, H. (2009). *J. Appl. Crystallogr.* 42, 339-341.
8. Guzei, I. A. (2007-2013). Programs *Gn*. University of Wisconsin-Madison, Madison, Wisconsin, USA.
